# The Roles of Tumor-Associated Macrophages in Prostate Cancer

**DOI:** 10.1155/2022/8580043

**Published:** 2022-09-07

**Authors:** Chenglin Han, Yuxuan Deng, Wenchao Xu, Zhuo Liu, Tao Wang, Shaogang Wang, Jihong Liu, Xiaming Liu

**Affiliations:** Department of Urology, Tongji Hospital, Tongji Medical College, Huazhong University of Science and Technology, Wuhan, China

## Abstract

The morbidity of prostate cancer (PCa) is rising year by year, and it has become the primary cause of tumor-related mortality in males. It is widely accepted that macrophages account for 50% of the tumor mass in solid tumors and have emerged as a crucial participator in multiple stages of PCa, with the huge potential for further treatment. Oftentimes, tumor-associated macrophages (TAMs) in the tumor microenvironment (TME) behave like M2-like phenotypes that modulate malignant hallmarks of tumor lesions, ranging from tumorigenesis to metastasis. Several clinical studies indicated that mean TAM density was higher in human PCa cores versus benign prostatic hyperplasia (BPH), and increased biopsy TAM density potentially predicts worse clinicopathological characteristics as well. Therefore, TAM represents a promising target for therapeutic intervention either alone or in combination with other strategies to halt the “vicious cycle,” thus improving oncological outcomes. Herein, we mainly focus on the fundamental aspects of TAMs in prostate adenocarcinoma, while reviewing the mechanisms responsible for macrophage recruitment and polarization, which has clinical translational implications for the exploitation of potentially effective therapies against TAMs.

## 1. Introduction

Statistics demonstrated that the morbidity rates of prostate cancer (PCa) have shown a remarkable increase worldwide, which seriously threatens public health and survival [1, 2]. Surgery and radiation are the standard primary treatment against early patients with localized prostate malignancies, followed by androgen deprivation therapy (ADT), like surgical or chemical castration if this disease recurs. Currently, sipuleucel-T, enzalutamide, abiraterone, and radium-223 have been approved by the Food and Drug Administration (FDA) for clinical application. Unfortunately, the response is only transient in initial treatments due to the intrinsic or acquired resistance, and thus has different effects on earlier endpoints and overall survival (OS) [3]. In addition, most patients will stop responding to ADT over a while and progress towards a lethal outcome known as castration-resistant prostate cancer (CRPC), which has not been adequately addressed [4]. Thus, prostate carcinoma remains a refractory malignancy, which wistfully calls for an original therapeutic strategy to lower the cost burden. There is also an unmet clinical need to further explore pathological mechanisms underlying carcinogenesis and progression.

It has been proved that tumorigenesis is a complicated and gradual process in which multiple mutations and progressive stages accumulate. Oncogenic mutation, like PTEN loss, is implicated in an early stage of prostate tumor development. Beyond tumor-intrinsic alteration, recent evidence points to the critical role of the tumor microenvironment (TME) in tumor progression and therapeutic response. TME, as a host of a sophisticated signal network, provides a fertile ground conducive to tumor survival [5]. Except for the subject of neoplastic cells, TME contains multiple nonmalignant stromal cells, like macrophages, endothelial cells, and fibroblasts [6, 7]. Among these cells, tumor-associated macrophages (TAMs) act as the central regulators of the interplay between tumor and surroundings [8]. In general, TAMs are divided into two dichotomous subsets: classically activated (M1) and alternatively activated (M2) macrophages [9], and their status depends spatially and temporally on integrated cues provided by TME [10]. Thus, this classification paradigm is an acknowledged oversimplification and could not accurately recapitulate native PCa-associated macrophages, since multiple macrophage subpopulations have been observed in vivo [10, 11].

Several researchers believe that aberrance in macrophage fate brings about a reverse clinical outcome, as in autoimmune disease and cancer. In metastatic-CRPC (mCRPC) samples, abiraterone or enzalutamide-sensitive patients exhibited increased pro-inflammatory mediators, including interferon-*γ* (IFN-*γ*), interleukin-5 (IL-5), and tumor necrosis factor-*α* (TNF-*α*), which were generally identified as M1 markers [12]. Macrophages have intrinsic tumoricidal properties, yet most TAMs predominantly displayed functional characteristics of M2-like macrophages in tumor sites [13]. Recently, we have come to appreciate that the M2-TAMs increased stepwise from normal prostate to mCRPC. A higher density of TAMs increases the risk of tumor recurrence after transurethral resection of the prostate [14]. The Gleason score (GS), a predictive index for disease progression, derives from a pathologist's evaluation of prostate cancer tissue microarrays. Basically, five different prognostic groups were classified according to the final score, when ≤6 points usually mean better prognostic results [15]. The abundance of M2-like macrophages is also strongly associated with a higher GS and indicates worse specific survival and recurrence-free survival after hormone therapy [16, 17]. In a large PCa cohort, patients with higher M2-TAM influx exhibited enhanced resistance to immunotherapy and had a nearly twofold increase in mortality [18].

The direct or indirect contact detail is the hotspot regarding cell-cell interaction. In this review, we first discussed the intrinsic mechanism responsible for TAM reprogramming and the effects of TAMs on PCa development in multiple aspects. Cancer immunotherapy strategies targeting TAMs are then presented.

## 2. Macrophage Origin

In addition to mediating the first line of defense against pathogenic insult, macrophages, as a crucial part of innate immunity, can repair damaged tissue to support tissue homeostasis [19]. The heterogeneity is a significant character of macrophages. Given that macrophages assume supportive functions specialized to their resident tissue compartments, they are endowed with different names, such as Kupffer cells (liver), Langerhans cells (skin), and osteoclasts (bone) [20]. Bone marrow–derived monocytes enter the bloodstream and reach the majority of the tissues in vivo, where they are further differentiated into tissue-resident macrophages. However, the histological macrophages also arise from an embryonic precursor (yolk sac and/or fetal liver). These macrophages appear to have stem cell–like abilities and persist throughout life by local self-renewal [19, 21]. Therefore, as in other tissues, macrophages in the prostate consist of both blood-derived and embryonic-derived populations, where it is still unclear whether macrophage lineages with distinct origins exert diverse functions.

Macrophages constitute the dominant population within the TME and can be identified and quantified by using CD68 staining as a marker [22]. The emerging evidence indicated it is a dual origin that TAMs derive from. In prostate tumors, the TME is preferentially enriched with myeloid cells in both human and murine models. Firstly, a variety of chemokines, like colony stimulating factor-1 (CSF-1), granulocyte colony stimulating factor (G-CSF), and C-C motif chemokine ligand 2 (CCL-2), whether secreted from tumor cells, or stromal host cells, are associated with the recruitment and formation of tumor-related immune cells. On the other hand, TAMs could originate from myeloid progenitors that exist in the yolk, as in glioma and pancreatic cancer [23, 24].

TAMs can be continuously replenished in vivo. A human PCa specimen reveals that a milieu containing abundant factors and vesicles derived from tumor cells drives TAMs to aggregate on the surface [25]. PTEN deficiency correlated with the CXCL8 upregulation and subsequent macrophage infiltration. RNA-sequencing showed that overproduction of chemokines is induced in macrophages, which may contribute to higher levels of myeloid cells in advanced prostate tumors. CCL2-CCR2 axis has been shown to modulate macrophage number and phenotype for TME remodeling, which is consistent with increased tumor volumes observed here. There is no difference in the proliferation of PC-3^lucCCL2^ and PC-3^lucMock^ in vitro. Nevertheless, the PC-3^lucCCL2^ tumor growth was significantly faster than the control in vivo. These data showed that CCL2 mediated the recruitment and retention of vast monocytic precursors in neoplastic tissues to enhance tumor burden, yet neutralizing antibodies targeting CCL-2 reduced macrophage mobilization [26, 27]. Also, spondin-2 (SPON2) overexpression has been observed in the serum or tissue samples of patients diagnosed as PCa [28]. Functionally, SPON2 activates PYK2 and increases its downstream RhoA and cortactin expression through interacting with *α*4*β*1 integrin, thereby promoting cytoskeletal remodeling of monocytes for transendothelial migration [29]. Colony-stimulating factor-1 receptor (CSF-1R), a primary regulator of macrophage development, correlates with normal prostate growth and prostate cancer progression. Human prostatectomy samples showed intense staining of CSF-1R in areas of carcinoma and TAMs.

A large number of myeloid cells infiltrated into irradiated tumor sites in a murine PCa model. Mechanistic studies have suggested that local irradiation induces ABL1-dependent CSF-1 production, followed by activation of CSF1/CSF1R signaling for systemic macrophage recruitment [30]. Strikingly, recent studies point to the intricate interaction of TAMs with stromal cells. CAVIN1 is abundantly expressed in the normal prostate stroma, while its level is downregulated in the PCa stroma. This decrease in stromal CAVIN1 contributes to the upregulation of inflammatory signatures, like increased matrix metalloproteinase-3 (MMP3), dickkopf-1 (DKK1), and CSF-1 secretion, thus attracting macrophages for a tumor-supportive microenvironment [31]. The urokinase-type plasminogen activator/urokinase-type plasminogen activator receptor (uPA/uPAR) axis may play a central role in the aggressive prostate disease through direct and indirect interactions with integrins, growth factors, and endocytosis receptors. To our knowledge, a study that demonstrated a direct link between activation of the uPA/uPAR axis and macrophage infiltration in PCa development has also been reported, in which stromal-derived uPA was regarded as a possibly predominant source within the TME [32, 33].

## 3. Macrophage Polarization

Except for their heterogeneity, macrophages are known to exhibit remarkable plasticity. The differentiated macrophages adopt appropriate phenotypes to regulate the diverse biological process. The functional evolution of macrophages is a highly dynamic process that is finely determined by signal transduction and metabolism [34]. The dichotomous classification of macrophages is currently generalized as the classically activated M1 and alternatively activated M2 phenotypes, which are described as extremes of functional states [35]. M1 and M2 macrophages represent distinct functions and transcriptional profiles in vivo, such as antigen expression, secreted factors, and metabolic pathways. The bi-directional activated potency of macrophages highly requires a key “switch” to respond to distinct peripheral stimuli. Macrophage destiny is not fixed, and two polarized states can be reversibly converted via reprogramming under a particular microenvironment. Indeed, macrophages exist across a dynamic spectrum, and even share mixed M1 and M2 characters.

In brief, M1 macrophages with phagocytosis property drive Th1 response and participate in the early inflammatory process. Lipopolysaccharide (LPS), an essential bacterial component, engages directly the Toll-like receptor 4 (TLR-4) on the membrane surface, thus enabling monocyte differentiation into an M1-like subtype that is characterized by higher levels of reactive oxygen species (ROS), inducible nitric oxide synthase 2 (iNOS), and MHC II molecules [36, 37]. Classical M1 macrophages execute pathogen clearance by secreting various pro-inflammatory factors, like IL‐1*β*, IL-12, and TNF‐*α*. Conversely, alternatively activated M2 macrophages mediate wound healing and fibrosis, thus leading to the resolution of inflammation. In the context of cancer, monocytes are quickly differentiated towards M2-like TAMs characterized by higher arginase-1 (Arg1) activity and the surface marker F4/80^high^ CD163^+^CD206^+^, suggesting a transition from L-arginine catabolism into depletion [38]. Supporting this notion, TAMs also highly express autocrine factors, like transforming growth factor-*β* (TGF-*β*) and IL-10, to promote their own maturation [39].

Generally, the most specific antibody recognizing CD163 could distinguish M2 from M1 macrophages. In the xenograft section of nude mice with seven common human PCa cell lines, more than 94% of all TAMs display an M2-like phenotype, and few M1-polarized macrophages are distributed in the periphery [17]. Recently, a study reports that prostate stereotactic body radiotherapy (SBRT), a neoadjuvant method to radical prostatectomy, amazedly alters the immune microenvironment within PCa. Multiplex immune-fluorescence (mIF) determines the increase of CD163^+^ macrophage subsets in densities and reaches a 5.61-fold change 2 weeks after SBRT [40]. M1-type polarization is experimentally induced in vitro using exogenous toll-like receptor agonist, IFN-*γ* or combined with LPS, whereas the most potent M2-type is attained upon Th2 cytokine (like IL-4 and IL-13) stimuli [41]. THP-1 monocyte from human peripheral blood is frequently used to study the impact of TAMs in carcinogenesis due to its reproducible nature. More importantly, molecular profiles of M1-/M2-subtype derived from THP-1 in vitro are similar to that in intracorporal macrophages that have undergone polarization [42].

**Figure 1:**
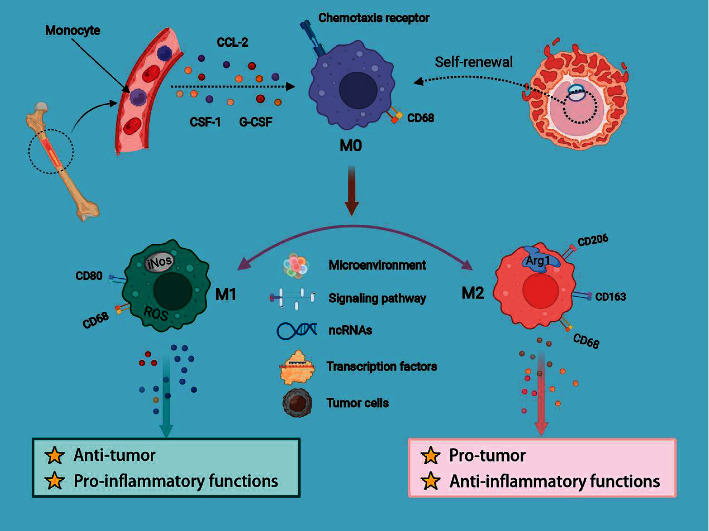
Representative images of the regulation of macrophage recruitment and polarization.

In view of the intricate signaling network in TME, the regulators of macrophage differentiation are not wholly revealed; thus, an enhanced understanding of their activation is helpful to identify effective molecular targets for pharmacological intervention. In this section, we emphatically illustrated the mechanism of macrophage polarization (Figure 1).

### 3.1. Microenvironment-Regulated Macrophage State

A variety of cytokines in the TME dictate the macrophage state. Macrophage colony-stimulating factor (M-CSF) appears competent to increase the number of TAMs through modulating GTPase Rac2, but M-CSFR blockers attenuate the polarization of Ly6C^hi^ monocytes to M2-like MHC-II^lo^ TAM [43]. Meanwhile, the extent of IL-6 expression in the stromal TME was positively associated with the abundance of F4/80^+^ TAMs in PCa specimens [44–46]. One study provides experimental evidence that IL-6 acts through the IL-6R/Janus kinase 2 (JAK2)/signal transducer and activator of transcription-3 (STAT3) pathway to skew THP-1 monocyte toward an M2-like phenotype, without affecting its migration [47]. In addition, IL-6 enhances IL-4-dependent M2 polarization by boosting IL-4R*α* expression under chronic inflammatory conditions [48]. An earlier study reported that the cross communication between cancer-associated fibroblasts (CAFs) and TAMs may further fuel PCa progression [49]. CAFs could induce inflammation and angiogenesis by stimulating macrophage infiltration via secretion of various cytokines, like monocyte chemotactic protein-1 (MCP-1), stromal-derived growth factor-1 (SDF-1), and CXCL14 [50]. In another study, CCR-2 dependent recruitment of macrophages by resident CAFs was reported to support tumor growth [51]. Recent data show that adenosine generated by CAFs can mediate the expansion and/or differentiation of M2-like macrophages [52]. Raised levels of G protein-coupled receptor 30 (GPR30) in prostate CAFs contribute to recruitment of monocytes and M2 differentiation of macrophage-like cells, which is partially associated with CXCL12 expression [53]. However, the staining of estrogen receptor alpha (ER*α*) in CAFs is relatively weak compared with the adjacent nonmalignant tissue, leading to a short time to hormonal relapse [54, 55]. Compelling evidence suggests that CAF.ER*α* (+) has a lower capability to attract macrophages into tumor sites and suppresses M2-type macrophages in the PCa microenvironment. After co-culture with the CAF.ER*α* (+), macrophages expressed less M2 macrophage-related markers, including IL-10, YM1 and Fuzz1, but not Arg-1. Further mechanism dissection indicated that CCL5 and IL-6 derived from CAF.ER*α* (−) may be implicated in macrophage recruitment and TAM generation [56]. Also, higher IL-4 and IL-13 levels in CAF.ER*α* (−) cells further support this conclusion.

Hypoxia-responsive macrophages favor fast tumor growth [57]. Hypoxia is a prominent characteristic of the TME, and an increase in M2-TAM infiltration is observed in prostate tumor tissues due to their tropism to hypoxia. Both 5-LOX and hypoxia inducible factor-1(HIF-1) in hypoxic areas boost macrophage mobility, partially by inducing MMP-7 expression [39]. Moreover, infiltrated macrophages tend to adopt M2-like features in the aged, oxidative, or mionectic milieu. One hallmark of the aging prostate is more significant infiltration of inflammatory cells, with a large release of growth factors. Compared with young mice, RM-9 prostate tumor cells orthotopically transplanted into the prostate grew at a faster rate in old mice. Further investigation showed that increased intra-tumoral leukocytes in the aged prostatic environment, especially F4/80^+^macrophage, could possibly be attributed to upregulation of unique cytokines profiles, including IL-6 and IL-9 [58]. Furthermore, hypoxia-mediated lower expression of intra-tumoral pigment epithelium-derived factor (PEDF) facilitates the transformation of monocytes into prostatic TAMs. Serving as an immune-modulatory factor, PEDF raises the levels of M1-specific markers, such as iNOS, IL-12, and TNF-*α*, whereas restraining IL-10 and Arg-1 expression in macrophages [59].

One critical metabolic nature of malignant cells is the enhanced activity of glycolysis, even in aerobic conditions, which is known as the “Warburg Effect” [60]. Lactate, metabolic by-products of glycolysis, forms a heterogeneous acidification niche that could direct the functional roles of macrophages. Lactic acids generated by glycolytic tumor cells were reported earlier to drive the pro-tumoral polarization of macrophages via the ERK/STAT3 signaling activation [61]. In line with these findings, recent studies show that lactate either mediates HIF-1 expression or increases Nrf2/heme oxygenase-1(HO-1) activation by elevating the intracellular ROS to elicit M2-like functional polarization [62]. Particularly in the late stage of PCa, elevated MCT4 expression is beneficial for preserving intracellular pH via assisting lactate outflow across the plasma membrane [63]. Current research proposes a scenario in which acidity, independent of lactate, can alter the activation state of TAMs. The zwitterionic buffer-based medium was used to simulate extracellular acidosis. Acidic condition (pH 6.8) did not affect the viability of activated macrophages, yet increased the expression of Arg1 and CD206 in IL-4-polarized macrophages. In addition, M2-like macrophage aggregation was usually accompanied by intra-tumoral lipid deposition. The fatty acid‐enriched TME induces mitochondrial respiration of infiltrating monocytes via modulating the mTOR pathway, thus regulating their pro-tumoral phenotype [64].

### 3.2. Signaling Pathway-Associated with Macrophage Education

Like the cyclic AMP (cAMP)‐protein kinase A (PKA) pathway, intracellular signaling cascades are responsible for M1/M2 polarization. Early research observed that both total PKA and phosphorylated PKA were decreased in the M1 subpopulation, whereas were upregulated in M2 subtypes. Mechanically, PKA regulatory II*α* subunit (PRKAR2A) can occupy the transmembrane domain of the IFN‐*γ* receptor to inhibit the downstream Jak2‐STAT1 pathway. On the other side, PKA provokes the activation of cAMP response element‐binding protein (CREB) for IL-10, Arg-1, and VEGFa generation, and amplifies the regulation of IL-4 on M2-type polarization [65]. Significantly, prostaglandin E2 (PGE_2_) promoted the activity of the EP4 receptor, thereby increasing anti-inflammatory signatures via a cAMP/PKA/CREB‐dependent pathway [66]. Paradoxically, though increasing PKA levels through interacting with EP4 receptor, PGE_3_ inhibits CD206 expression in THP-1 cells and reduces the proportion of CD68^+^ and CD206^+^ cells in tumor tissue. According to a recent study, there is only a slight but stable increase of PKA in the M2-like phenotype, yet PKA inhibitor H‐89 can suppress a shift towards the M2 state. It is plausible to speculate that M2-TAM polarization depends on PKA activation that is reversely insufficient for M2 skewing, which warrants further investigation into the significance of PKA in macrophage differentiation [67].

LAMP2a (lysosome-associated membrane protein type 2A), located in the lysosomal membrane, acts as a novel intracellular switch to re-educate macrophages towards M2 phenotype through selectively targeting and degrading substrates of CRTC1 and PRDX1. At tumor sites of LAMP2a KO mice, an increase in pro-inflammatory M1-like subtype was observed. LAMP2a is manifested to be activated by various external stressors in TME, such as hypoxia and androgen deprivation [68]. The RON receptor (MST1R), a Met tyrosine kinase receptor family member, exists preferentially in prostate epithelial cells and macrophages [69, 70]. Prior work demonstrated macrophage-intrinsic RON as a negative regulator of macrophage activation. Specific loss of RON signaling in macrophages increased intra-tumoral iNOS staining in the transgenic adenocarcinoma of mouse prostate (TRAMP) model. Notably, prostate epithelial RON heightens MST1R activity of macrophages in a paracrine manner and promotes IL-6 and IL-33 generation, which indirectly accelerates prostate tumor growth through driving M2-like macrophage polarization [71]. However, macrophage scavenger receptor (MSR) is only expressed in macrophages, and MSR-positive inflammatory cells are broadly considered as M2-subtype in several types of malignancies, like glioma and ovarian epithelial tumors. In contrast, a sequence deficiency of MSR was initially found in one metastatic PCa sample, and the decreased expression of MSR was a predictor of poor prognosis [72, 73]. Immunohistochemistry analysis suggests that MSR labels most M2-macrophages in the PCa biopsy. IL-6/TGF-*β* restricts the MSR-transcriptional levels of THP-1 cells, thus modifying the gene expression of M2 markers. It probably ascribes to the comprehensive outcome of multi-factors in vivo, like distinct lineages, surrounding stromal cells, and the local environment, and the role of MSR in macrophage polarization remains to be further elaborated [74].

TGF-*β*, as a pleiotropic cytokine, exerts dual functions in cancer. Flow-cytometric analysis shows that recombinant TGF-*β* upregulates the expression of M2 markers in THP-1 cells, which points to the involvement of the TGF-*β*-mediated pathway in the regulation of macrophage phenotypes [75]. TGF-*β* induces M2-like polarization through Snail mediation, where downstream SMAD2/3 and PI3K/AKT signaling activation is indispensable [76]. TGF-*β* also synergizes with IL-10 to enhance the activation of M2 macrophages [77]. One novel finding that the activated Akt/FoxO1 pathway induced by TGF-*β* is responsible for the transformation of LPS-stimulated macrophages toward M2-subtypes improves our understanding of the roles of TGF-*β* in M2 polarization [78]. The bone morphogenetic protein (BMP) is a pivotal member of the TGF-*β* super-family, and specific deficiency of myeloid BMPR1 leads to an increase in the number of TNF*α*^+^ M1 macrophages, thus impairing mouse prostate cancer growth [79]. Various BMP ligands, like BMP4, BMP6, and BMP7, can support M2 macrophages, whereas the BMP inhibitor DMH1 impedes M2-like polarization of macrophages isolated from tumors, with lower IL-10 and Cox2 levels. These findings supported our hypothesis that BMP signaling is noteworthily required for M2 macrophage activation [80].

### 3.3. Noncoding RNAs (ncRNAs)-Mediated Epigenetic Regulations

Noncoding RNAs are a unique subclass of regulatory RNAs that cannot be translated into proteins. MicroRNA (miRNA) and long noncoding RNAs (lncRNA), as master epigenetic molecules, could regulate about 90% of human genes, which has attracted more attention. Among them, lncRNAs with a length greater than 200 nucleotides can be either cis or trans acting element to regulate gene expression in all respects: epigenetic, transcriptional, and posttranscriptional. lncRNAs, as molecular signals, decoy, guide, or scaffold, interact with DNAs, mRNA, other ncRNAs, or protein to exert their functions [81]. miRNAs are characterized by a length of about 22 nucleotides and control cellular biological processes by pairing to specific sequences of diverse mRNAs 3′untranslated region (3′UTR) at the same time [82]. Several ncRNAs and proteins have long been believed to play pivotal roles in determining the direction of macrophage polarization [83]. Lnc-M2 binds to PKA protein to activate its downstream CREB, thereby facilitating the process of M2 macrophage polarization [84]. Previously, a study indicated that M1 macrophages had higher levels of lncRNA-CCAT1 than that in M0 and M2 macrophages. Knock-down of CCAT1 increased the M2-phenotypic transformation and subsequent pro-tumorigenic functions by regulating miR-148a/PKC*ζ* [85]. However, LINC00467 favors the higher expression of M2-characteristic genes via the miR-494-3p/STAT3 axis in prostate cancer [86]. Similarly, lncRNA-SNHG1 promotes M2-like macrophage polarization via increased STAT6 phosphorylation [87]. In macrophages, lncRNA-EPS is defined as a transcriptional brake that inhibits pro-inflammatory gene expression. Mechanistic studies indicate that lncRNA-EPS interacts with heterogeneous nuclear ribonucleoprotein L (hnRNPL) via a CANACA motif located in its 3′ end to change nucleosome position and repress transcription of immune response genes (IRGs) [88]. LncRNA CDKN2B-AS1 is principally expressed in the nucleus of THP-1 macrophages and restrains M2 polarization by forming RNA-DNA triplex with CDKN2B promoter [89]. miR-101-3p drives a pro-inflammatory phenotype in unpolarized monocyte-derived macrophages (MDMs), at least in part by targeting TRIB1. qRT-PCR results demonstrated that miR-101-3p dramatically elevated the mRNA levels of TNF-*α*, IL-8, and CD80, whereas having no effects on the expression of M2-associated genes [90]. As an inhibitory regulator of JAK/STAT signaling, suppressor of cytokine signaling (SOCS) negatively regulates the state of macrophages and dendritic cells [91]. miRNA let-7b-5p overexpression domesticates macrophages towards M2-subtypes through regulating the SOCS1/STAT pathway, followed by prostate cancer progression [92].

Circular RNAs (circRNAs) are single-stranded RNAs characterized by having covalently closed loops and specific tertiary structure. Current studies support that circRNAs function as miRNAs sponges, alternative splicing mediators, and protein templates [93]. A high throughput circRNA microarray assay was conducted to evaluate circRNA signature of M1 and M2 macrophages. Compared with M2 macrophages, the expression of circRNA-010056, circRNA-003780, and circRNA-010231 is upregulated in M1-type macrophages, whereas the levels of circRNA-013630, circRNA-003424, circRNA-018127, and circRNA-001489 are downregulated [94]. circPPM1F is involved in LPS-induced M1-like activation through forming circPPM1F–HUr–PPM1F–NF-*κ*B axis [95]. circCdy can also mediate M1 polarization by curbing the transportation of IRF4 into the nucleus [96]. However, circSAFB2, as a sponge for miR-620, promotes the polarization of M2 macrophages through modulating JAK1/STAT3 axis [97].

### 3.4. Transcription Factors

Directed by extracellular signals, several transcription factors, including interferon regulatory factors (IRFs), NF-*κ*B, c-Myc, STAT3/6, Klf4, and C/EBP*β*, participate in cellular transformation. TLR-4-mediated activation of downstream IRFs and NF-*κ*B regulators facilitates iNOS expression and NO generation, yet deletion of c-Myc or C/EBP*β* in macrophages impairs M2-like programs [98]. c-Myc is not implicated in macrophage proliferation and survival, but it is pivotal in alternative macrophage activation. c-Myc controls 45% of M2-related genes by either directly interacting with their promoters (e.g., MRC1 and ALOX15) or indirectly influencing other transcription factors (e.g., STAT6 and PPAR*γ*) [99]. In c-Myc-KO mice, isolated TAMs exhibit attenuated abilities of tissue remodeling [100]. Furthermore, myeloid cells undergo M2-phenotypic alteration under FBXW7 knockout conditions. Mechanistically, FBXW7 deficiency attenuates the K48-linked polyubiquitination and resultant degradation of c-Myc, followed by higher levels of Arg1, Ym1, and Fizz1 [101].

STAT3 positively affects the phenotypic transition from M1 into M2 mediated by prostate tumor cell-culture supernatant. JAK2/STAT3 signaling activation is implicated in IL-6-mediated M2-like polarization. In addition, p-STAT3 encourages M2 activation through inhibiting negative regulators, such as NF-*κ*B and p-STAT1 [102]. Cooperation between STAT6 and PPAR*γ* is responsible for IL-4-orientated M2-polarization, whereas STAT6 acetylation is a negative regulatory mechanism underlying M2 polarization. The E3 ligase Trim24 catalyzes CREB-binding protein (CREBBP) ubiquitination and subsequent STAT6 acetylation to restrict M2 macrophage activation [103]. Notch signaling, a highly conserved pathway, is recognized as the determinant in the orientation of macrophage polarization. When the Notch transduction is blocked, macrophages exhibit functional characteristics of an M2-like phenotype. However, in the absence of myeloid Klf4, bone marrow–derived macrophages (BMDMs) express fewer M2-like indicators, like Arg-1, mannose receptor (MR), and display a pro-inflammatory gene expression signature supporting M1 differentiation [104].

Androgen receptor (AR), as a member of the nuclear receptor super-family, is involved in regulating cellular events. A study illustrates that activation of AR signaling is not exclusively limited to prostate epithelial cells but lies in TAMs. AR translocates into the nuclei and binds DNA at enhancer regions via the AP-1 complex in macrophages, which is incompatible with epithelial tumor cells where AR acts on the DNA by transcription motifs. ChiP-seq analysis indicated AR-binding proximal to M2-symbolic genes of tissue-resident macrophages, including IL-10, CD163, and CD206. Androgens have broad immune-regulatory effects, and androgen/AR may exert an unexpected role in macrophage polarization. These genes were elevated upon R1881 stimulation, whereas RD162, an AR signaling blocker, partially restored the initial expression. The reconstitution of androgen (DHT, dihydrotestosterone) also significantly increased the relative percentage of CD163^+^CD206^+^ double-positive in MDMs. The M2-promoting effect of DHT was just specific for macrophages via AR-binding sites that were most prominently found in intronic and distal intergenic regions. Interestingly, bicalutamide and flutamide reduced CD163^+^ macrophage infiltration, while promoting the expression of pro-inflammatory cytokines (IFN-*γ*, TNF-⍺) in PCa samples; however, later studies reported contradicting results [105, 106].

Furthermore, a trend towards fewer TAM markers was observed in alveolar macrophages lacking AR compared to AR-proficient macrophages [107]. Some investigators found that IL-4 initiates AR signaling for M2 differentiation, supporting AR as an enhancer of TAM differentiation [108]. Indeed, AR expression in macrophages from endogenous sources is deficient, less than 100 times than in PCa cell lines. Given that ADT is a systemic treatment, the simple inhibition of AR signaling by ADT may be insufficient for transformation from M2 toward M1 phenotype. Generally, ADT domesticates the M2-like polarization in a paracrine pattern by modulating other host cells, including tumor cells, which are not only limited by blocking the androgen/AR axis in macrophage-like cells.

### 3.5. Tumor Cells

A study indicates that human macrophages undergo certain alterations in the presence of PCa cells, which is not easily surmounted. Prostate TAMs could be reprogrammed through direct contact with PCa cells. Till date, multiple lines of evidence consistently indicate that there is a paracrine effect between cancer-derived factors and recipient macrophages. Yet, these immunomodulatory mediators certainly warrant investigation into their specific contributions to macrophage activation.

The supernatant of PC-3 cells effectively leads to a change in macrophage profile from M1 into M2 in vitro, and that shift is mainly associated with IL-10-mediated STAT3 phosphorylation. Similarly, milk fat globule-EGF factor 8 (MFG-E8) contained in exosomes from PCa cells drives an M2-like state by activating the STAT3/SOCS3 pathway [109]. A basic research yields interesting discoveries that PCa-derived CRAMP firstly chemoattracts immature myeloid progenitors (IMPs) into the TME and then modulates their differentiation into the M2-like macrophages. Molecular mechanisms indicate that CRAMP upregulated formyl peptide receptor 2 (FPR2) in an autocrine pattern, thereby inducing STAT3-dependent M-CSF and MCP-1 generation for M2 skewing in CRAMP-enriched TME [110]. Protein kinase C zeta (PKC*ζ*), a tumor suppressor, negatively correlates with the abundance of CD206^+^ macrophages. A co-culture model is used to simulate the physiological interaction between PCa cells and macrophages herein. Silencing of PKC*ζ* in PC-3 and DU145 cell lines indirectly initiates M2 polarization by mediating the secretion of critical cytokines, including IL-4 and IL-13 [111]. In addition, the generation of TNF-*α*/*β* and M2 macrophages was attenuated in the PC3-shKPNA4 primary tumor tissues. Comprehensive analysis suggested that TNF activated by KPNA4 increases TAM gene markers in primary mouse monocytes, altering the microenvironment for immune escape [112]. However, another study demonstrated the positive correlation of TRIB1 with the frequent presence of CD163^+^ macrophages in clinical PCa specimens [113]. Mechanistic dissection revealed that TRIB1 contributed to the secretion of CXCL2 and IL-8 via IKB-zeta mediation in tumor cells, followed by an increase in the M2-like population. Furthermore, enhanced AIRE regulated by transcription factor Elk-1 in androgen-independent PCa cells is equipped to polarize peripheral monocytes towards an M2-like phenotype by modulating IL-6 and PGE secretion [114].

Tumor cells also control the macrophage-activated state by indirectly influencing/activating peripheral stroma cells within TME. Macrophage inhibitory cytokine‐1 (MIC-1) production is augmented in prostate cancer cells by adipocytes-mediated lipolysis and fatty acid release, which enforces the secretion of IL-6 and IL-8 from periprostatic CAFs for M2-polarization [115]. Macrophages can undergo phenotypic transformation via metabolic reprogramming. Alternatively activated macrophages have been shown to prefer fatty acid oxidation [116]. Notably, KRAS^G12D^ is generated in tumor cells via autophagy-dependent ferroptosis in the oxidative microenvironment and is subsequently packaged into exosomes for release. Extracellular KRAS^G12D^ is taken in by macrophages via AGER/RAGE, followed by M2 activation via STAT3-dependent fatty acid oxidation [117].

## 4. The Roles of Macrophages in Tumorigenesis

**Figure 2:**
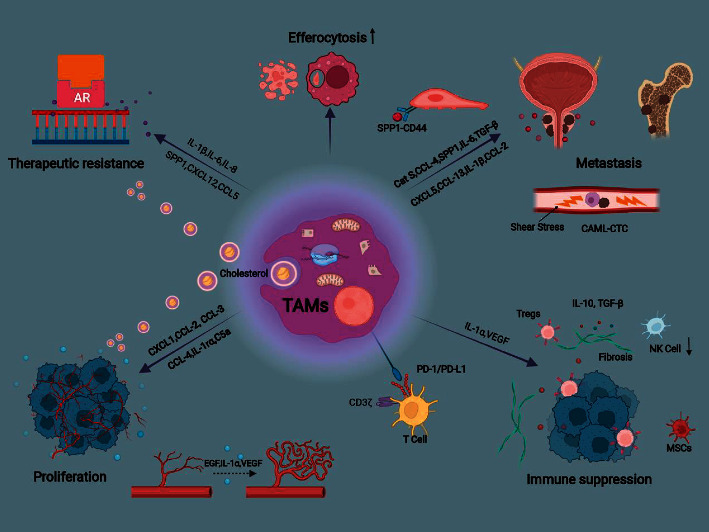
Tumor-associated macrophages mediate therapeutic resistance, proliferation, metastasis and immune suppression in prostate cancer. TAMs, tumor-associated macrophages; cat (S) cathepsin; CAML, cancer-associated macrophage-like cell; CTC, circulating tumor cell; tregs, regulatory T cells; NK, nature killer; MSCs, mesenchymal stem cells.

The transition from premalignant lesions to adenocarcinoma is a multistep process. It is commonly accepted that TAMs, as highly active immune effectors, exhibit either antitumor or protumor activity, which hinges on tissue-specific regulation and tumor-developed stage. Chronic inflammation is an epidemiologic factor for prostate cancer. In nascent tumors, circulating precursors are recruited to gather around the inflammatory milieu, and are subsequently differentiated into M1-like macrophages. They secrete stimulatory cytokines activating T effector cells to eradicate mutant neoplastic cells. Conditional medium (CM) of M1-polarized macrophages promotes apoptosis of PC-3 cells and suppresses tumor parameters, including metastasis and angiogenesis [118]. Nevertheless, a large number of M1 macrophages can also aggravate inflammatory damage in various pathological processes. Massive M1 activation causes severe cytokine storms and corresponding mutation accumulation in prostate epithelial cells via repeated injury, ultimately initiating the carcinogenic process. Complex and dynamic communication occurs between cancer cells and immune cells. As tumors grow and spread, the macrophage statue is subverted towards an antiphlogistic phenotype, representing alternation of the immune compartments. Advances in cancer research coincidentally suggest that M2-macrophages make up the majority of TAMs, especially in advanced stages of PCa, which usually correlates with an increased lethal risk [10, 119, 120]. The biological roles of TAMs in tumor formation and progression are multifactorial, which will be reviewed below (Figure 2).

### 4.1. Therapeutic Resistance

Endocrine therapy is the primary treatment for patients with advanced PCa. Nearly all patients still inevitably progress to advanced CRPC after about two years, which remains a major clinical challenge, despite primary symptomatic relief. Our initial efforts focused on exploring cell-autonomous alteration in CRPC populations. lncRNA HOXD-AS1 regulates chemoresistance of CRPC via WDR5 recruitment [121]. Activation of MAPK signaling by CXCR7 contributes to enzalutamide resistance [122].

Multiple studies emphasized that TAMs influence the clinical response to hormone therapy in vivo. Patients with TAMs <22/high power field (HPF) assume a better response to ADT than those with higher numbers of TAMs (*P* < 0.001). Currently, we notice that a decrease of M2-like macrophages mediated initially by androgen deprivation is transient and appears to reverse with long-term ADT employment [123]. Castrating tumor-bearing mice triggers an influx of leukocytes into prostate tumors. The increased stained intensity of CD68 and CD163 has been described in castrated TRAMP mice and radical prostatectomy samples from ADT-treated patients. SEMA3A was co-expressed with TAMs and correlated with the progression of CRPC. Interestingly, the SEMA3A transcription levels are upregulated after ADT, which elicits recruitment and M2-like polarization of monocytes via NRP1 receptor and promotes ADT resistance [124].

Critically, results from in vivo and in vitro models confirm that the emergence of CRPC partially depends on the production of compensatory growth factors by TAMs. Macrophages were also demonstrated to stimulate AR translocation into the nucleus in PCa cells in co-cultures. IL-1*β*, primarily generated in the M2-subtype, either mediates MEKK1 activation or causes TAB2-dependent nuclear receptor corepressors (N-CoR) dismissal from AR, which contributes to therapeutic resistance via conversion of AR antagonists to agonists [125]. Persistent AR activation remains a critical driver in castration resistance. Despite castrated levels of serum androgens, there is a higher degree of intra-tumoral androgens in CRPC, nearly the same as those of eugenic men [126]. Prostate cancer cells express most steroidogenic enzymes and are therefore capable of converting cholesterol to androgens. Single-cell RNA sequencing analysis shows that this subset of TAMs is characterized by an accumulation of lipids. Clear evidence points to an appreciable increase in the transcriptional levels of steroid and bile acid in M2-like TAMs.

BMDMs can express various genes associated with cholesterol influx/efflux more than the PCa cell lines, including Abcg1, CD36, and Scarb1 [127]. They could absorb cholesterol in the form of low-density lipoprotein (LDL) and transfer particles containing rich cholesterol into neighboring prostate tumor cells, where it acts as a precursor to enhance androgen biosynthesis for nuclear translocation of AR [128]. The BMP-6 secreted by PCa cells induced IL-6 generation in recipient macrophages, and reciprocally, the AR-transcriptional activity was elicited to avail castration resistance in CaP cells [129].

Alternatively, PCa progression may arise in the deficiency of a functional AR. As the strategies targeting AR have become widespread, the incidence of neuroendocrine prostate cancer (NEPC) has risen substantially, which manifests with lower AR signaling activity and grows independently of the androgen. Neuroendocrine differentiation (NED) is an emerging mechanism of resistance to cancer therapies [130]. NEPC cells themselves acquire the characteristics of stem cells, while conducing to resistance acquisition by surrounding tumor cells [19]. IL-6, a pleiotropic cytokine, activates the TGF-*β*/SMAD2 axis and its downstream p38MAPK to drive NED of PCa cells under androgen depletion conditions [131]. IL-6 derived from TAMs also appears competent to upregulate the expression of parathyroid hormone-related peptide (PTHrP) that is an indicator of NED, which forms a positive loop and leads to enhanced NEPC tumorigenesis in vivo [132]. Noteworthily, sterol regulatory element-binding protein−2 (SREBP-2) increased tumor-synthetic cholesterol, which can be assimilated by TAMs via scavenger receptor (SR)-mediated endocytosis for IL-8 generation [133]. Increasing evidence suggests that IL-8 can promote the NED of prostate cancer through activating MAPK/ERK signaling. It is also verified to attenuate TRAIL-induced apoptosis of PCa cells by regulating c-FLIP transcription [134].

SPP1 has great significance to M2 polarization and phenotype maintenance [102]. Single-cell data identifies SPP1 as a luxuriant TAM-secretary factor under the hypoxic microenvironment. SPP1 either expands the glycolysis program or increases the expression of p-glycoprotein for multidrug resistance in PCa cells [135]. Since the reciprocal crosstalk formed by TAMs with tumors, the second-line treatment like docetaxel combined with prednisone may also fail to restrict the aggressiveness of advanced CRPC. With exposure to docetaxel, the levels of several cytokines secreted by PCa cells were increased, like CSF-1 and IL-10, thus counteracting its anti-cancer efficacy [136]. Especially, CSF-1 stimulates polarization of neighboring macrophages towards an M2-like phenotype that reciprocally releases CXCL12 to sustain tumor survival via CXCR4 [137]. Meanwhile, TAM-derived CCL5 activates *β*-catenin/STAT3 signaling and upregulates the transcription factor Nanog, thus resulting in increased chemoresistance of PCa [138, 139].

### 4.2. Proliferation

TAMs can modulate early prostate tumorigenesis by promoting genetic instability, independently of any other carcinogenesis [140]. AR activator CCL-4 generated by M2-polarized macrophages mediates downregulation of P53/PTEN in RWPE-1 cells and augments the production of EMT markers. All prostate disorders are initially attributed to excessive proliferation of normal prostate epithelial cells. A 3-D co-culture model reveals that an elevated proliferative rate of normal prostate PZ-HPV-7 epithelial cells is achieved via activation of ERK and AKT induced by TAMs-secreted cytokines, like CCL-3, IL-1ra, and GDNF [25]. In another experiment, immortalized RWPE-1 cells alone formed well-organized spheroids after 24 days, whereas aggregated into a disorganized structure when cultured with macrophage-CM. Prostate intraepithelial neoplasia (PIN), a precursor lesion, is always the first step for prostate tumor construction [141]. TAMs are capable of increasing the percentage of nuclear cyclin D1-positive PIN cells. Later tests demonstrated that CXCL1, C5a, and CCL-2 derived by TAMs mainly potentiate PIN-cell proliferation through activating ERK without impacting cell apoptosis [142]. High-fat diet (HFD)-accelerated tumor growth was correlated with the increased M2/M1 ratio and IL-6 expression in the model mice. In human prostate cancer, IL-6 secretion was restricted to the prostatic stromal component, whereas IL-6 was derived mainly by local macrophages upon HFD stimulation. Higher IL-6 levels result in prostate cancer progression via STAT3 phosphorylation in tumor cells [143]. TAM-released IL-8 was demonstrated to sufficiently drive prostate tumor formation by modulating the STAT3/MALAT1 axis [144].

Given that tumor growth requires nutrition, the pro-angiogenic properties of TAMs are considered as the culprits of malignant transformation. To support this, a histopathologic study shows that the number of CD163^+^ macrophages is positively associated with the micro-vessel density (MVD) and proliferative Ki67-stained intensity [145]. TIE-2^+^ TAMs exhibit a unique angiopoietin receptor with neovascular capacity. Furthermore, M2-like TAMs secreted epidermal growth factor (EGF) to actuate neovascularization and carcinogenesis. The Smad1-induced IL-1*α* production of macrophages is an important mechanism whereby BMP-6 accelerates prostate tumor growth in vivo [146]. IL-1*α*, known as pro-angiogenic chemokines, enhances PCa-associated angiogenesis via IL-1R/CXCL8 [147]. Similarly, CCN3-mediated M2 phenotype increased VEGF expression and subsequently triggered endothelial tube formation. A pre-clinical study observed the elevation of ER*α* in prostate tumor mass and cells. It simultaneously demonstrated that ER*α* activation initiates downstream oncogenic signaling by interacting with HIF-1*α* in the hypoxic milieu [148]. Cholesterol from TAMs also acts as an endogenous ER*α* agonist to favor adaptive growth of prostate cancer. Recently, TAM is also identified as a crucial cellular link in paracrine tumor-tumor interplay. Consistent with animal experimental results, high-grade tumors encouraged the rapid growth of adjacent less-malignant tumors in patients with multifocal prostate cancer [149]. However, high-metastatic MLL did not affect low-metastatic AT1 viability in a co-culture system, suggesting that direct interactions between tumor cells were minor. Further analysis shows that soluble factors derived from an aggressive prostate tumor circulate into the milieu of distant indolent tumors, where neovascularization for blood supply markedly increases due to the massive accumulation of M2-like TAMs.

### 4.3. Metastasis

In prostate cancer, an increased proportion of iNOS^+^ macrophages is observed in organ-confined foci. In contrast, higher infiltration of CD163^+^ macrophages is conducive to extracapsular extension, suggesting M2-type macrophages can increase PCa-metastatic potential [50, 119]. IL-4 is responsible for the generation of macrophage-supplied cathepsin S (Cat S) protease that heightens the risk of pelvic metastasis in TRAMP mice and patients with prostate malignancy [150, 151]. Perivascular Cat S pro-form is activated in an acidic condition, allowing further E-cadherin degradation during metastatic colonization of distant organs. Secreted SPP1 remodel extracellular matrix for prostate cancer invasion [28]. The interaction of SPP1-CD44, a cell-surface receptor, is an important paracrine pattern to regulate tumor metastasis. Single-cell analysis also suggests that EMT is the biological process in tumor cells most relevant to SPP1^+^TAMs [152]. It was previously established that AR activation directly controlled the transcription of Triggering Receptor Expressed on Myeloid cells-1 (TREM-1) and upregulated its downstream chemokines, such as CCL-3, CCL-4, and CCL-13, which nonspecifically bind to chemokine receptors to promote the exfoliation of PCa cells. Immunohistochemical staining showed that CCR2, CCR3, and CCR4 staining were observed in primary and metastatic PCa cells on tissue microarrays, while CCR3 and CCR4 were absent in normal prostate cells [105]. Also, the elevation of CCL-4 is associated with Snail upregulation in high-grade PIN and prostate carcinoma [140].

Paradoxically, ADT increases the probability of distant metastasis in some PCa patients, although accompanied by tumor decrease and reduced PSA levels [104]. A clinical trial shows that 52% of PCa patients develop new bone lesions after abiraterone treatment for four months, which may be at least partially explained by increased TAM-secretory cytokines. Inquiringly, targeting AR with siRNA in macrophages promoted the migration of LNCaP cells in a co-culture system. Further analysis found that AR silencing induced CCL-2 elevation and resultant EMT process of PCa cells via CCL2/CCR2/STAT3 signaling [153]. Bone metastasis commonly occurs in men with recurrent CRPC, leading to a 5-year survival rate of 25%. M2-like TAMs constitute one-sixth of the total cells in PCa-resident bone lesions. Flow cytometry demonstrated that a vast majority of CD163-positive cells exist in mCRPC tissue obtained from the first right rib. Double-staining showed that HO-1 is broadly produced in CD163^+^ cells, and its expression is higher in aggressive prostate tumors. HO-1 is found to facilitate iron delivery and Fe^3+^ accumulation in tumor cells, whereas inhibition of HO-1 significantly retards bone-metastases in other PCa experimental models [154]. Ca2+/calmodulin (CaM)-dependent protein kinase kinase 2 (CaMKK2) is selectively expressed in macrophages. It regulates metabolic responses and manipulates the niche of bone microenvironment to benefit PCa cells by releasing inflammatory cytokines [155]. Both CCL-2 and IL-6 secreted from TAMs are also demonstrated to add a risk of bone metastasis, thus determining the advanced stage of metastatic prostate tumors [26, 107]. Instead, cancer cell death could lead to tumor growth and bone destruction, which partially ascribes to macrophage-efferocytotic capacity. During tumor progression, chronic inflammation inevitably causes tissue damage and cell death. Apoptotic/necrotic tumor cells are cleared and phagocytosed by macrophages, known as efferocytosis. Efferocytosis is crucial to preserve tissue integrity but may also have deleterious effects. Compared to M1-like macrophages, M2-like macrophages were displayed to be ∼4-fold more capable of efferocytosis. A study reported that efferocytosis of TAMs induces CXCL5 secretion by activating NF-*κ*B and STAT3 signaling in vitro, thus accelerating colonization of disseminated PCa cells and osseous progression [156]. In the context of cancer, the efferocytotic function of TAMs could be further enhanced by chemotherapies or other targeted therapies. This amplificatory effect triggers the secretion of extensive pro-inflammatory cytokines, like TGF-*β* and CCL-2, which perpetuates M2 polarization and forms feed-forward loops to exacerbate skeletal metastasis [56].

The metastatic process of prostate tumors is accompanied by prominent alteration in the basement membrane and extracellular matrix (ECM). Much evidence depicts that intense MMP-9 and IL-1*β* staining were observed in M2-like macrophages and tumor cells at the invasive prostate zone. MMP-9, as a cancer biomarker, participates in ECM degradation and facilitates tumor aggressiveness. Emerging roles of IL-1*β* in PCa development have been revealed. IL-1*β* activates AR function for enhanced tumor mobility [157]. Besides, a raised level of MIC-1 mediated by IL-1*β* in serum contributes to actin reorganization of tumor cells through activating FAK-RhoA signaling, thus reducing adhesion at an early stage [158]. PCa cell-derived IL-1*β* promoted Marco-dependent lipid accumulation, and reciprocally, the migratory capacity of tumors was enhanced by CCL6 released by lipid-loaded TAMs [159]. Nowadays, several studies emphasize that TAM-derived uPA mediates uPAR-dependent cleavage of the *α*6*β*1 integrin (*α*6p*β*1), which means pericellular laminin proteolysis [33].

Aggressive tumor cells intravasate into the blood vessel, thus becoming circulating tumor cells (CTCs). Homing to target organs is only possible when PCa cells survive in the circulation. Recently, a study reported that TAMs leave primary sites and attach CTCs in the peripheral blood of PCa patients, which is called circulating cancer-associated macrophage-like cells (CAMLs) [160]. Further investigation shows that contacts with CAMLs induced epithelial-mesenchymal plasticity and endow CTCs with an aggressive nanomechanical phenotype that can resist the shear stress of the bloodstream and advance seed-distant metastases [161].

### 4.4. Immune Suppression

The M1/M2 imbalance skews the immune response to opposite directions, which weakens immunological monitoring and helps tumor cells avoid the lethal attack. Macrophages of M2-like subtype themselves have poor antigen-presenting nature and allow desensitization of PCa cells to cytotoxicity mediated by nature killer (NK) cells. Several indirect and direct actions of TAMs on autologous T lymphocyte number and activation have been suggested. The depletion of L-arginine, an essential nutrient for T cells, is a vital contributor to the immunosuppressive TME in patients with cancer. Increased Arg1 expression in M2 macrophages could induce metabolic starvation of effector T-cell by clearing L-arginine, which favors the lower frequencies of circulating cytotoxic T cells. mCRPC lesions, known as “cold” tumors, exhibit poor T-cell infiltration and functionally inactive T cells. A recent study illustrated that TAMs either exclude CD8^+^ T cells from tumor mass via granulin-induced fibrosis or impair T-cell activities by inhibiting its-receptor CD3*ζ*chain, thus aggravating the immune evasion to support unchecked neoplastic growth [10, 162]. PD-L1 expressed on TAMs also binds to PD-1 of T cells, and subsequently transmits inhibitory signaling into T cells [163]. More significantly, TAMs phagocytose T cell-superficial anti-PD-1 antibodies and lower therefore the benefit from immunotherapy [164]. Regulatory T cells (Tregs) control cellular responses to death-associated stimuli by affecting both innate and adaptive immunity in the context of cancer. They can inhibit the activation of CD4^+^, CD8+ T cells, and NK cells via cytokine generation (IL-10 and TGF-*β*) or direct cell-cell contact, thus operating immunosuppressive functions. High Treg numbers have been described in the peripheral blood and tumor mass of PCa patients, which is why the vaccine has a weak antitumor effect [165]. Bioinformatics analysis points to a positive association of TAMs and Tregs in number in high-risk score PCa patients [119]. TAMs may induce Tregs production and cooperate with them to form a unique niche that elicits tumor progression. Elevated expression of Axl, MerTK, and Tyro3 receptor kinases acquired in M2 macrophages stimulate the influx of lymphocytes and its succedent differentiation into Tregs [12, 166]. Strikingly, TAMs assist mesenchymal stem cells (MSCs) in displaying enhanced immunosuppressive activity in PCa. IL-1*α* derived from TAMs induces excessive expression of TGF-*β* in MSCs [167]. Likewise, TAMs-released VEGF restrains the maturation of dendritic cells and increases the proportion of MSCs, thereby influencing the overall organization of the immune response [168].

## 5. Promising Strategies for PCa Treatment via Targeting TAMs

**Figure 3:**
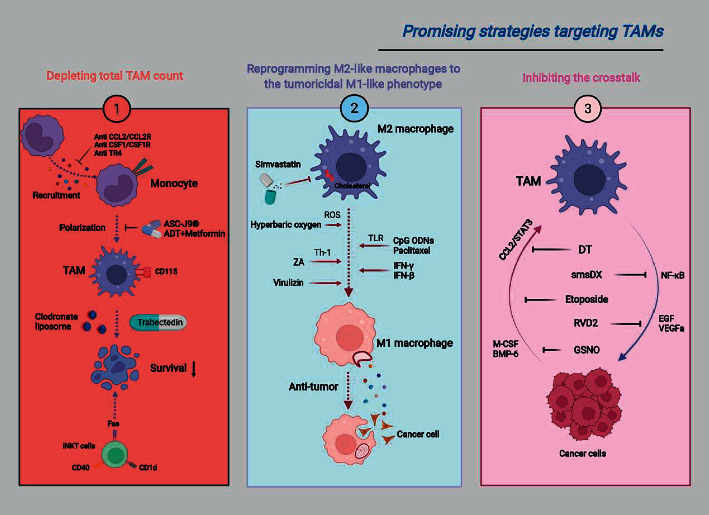
Promising strategies targeting TAMs for prostate cancer therapy. DT, dihydroisotanshinone I; ZA, zoledronic acid; smsDX, somatostatin derivate; RVD2, resolvin D2.

Given the dual roles of TAMs in orchestrating PCa progression, significant attention has been drawn to tumor immunotherapy targeting TAMs to disrupt immune tolerance. Broadly, current TAM therapeutic strategies pertain to the following three groups: (1) depleting total TAM count; (2) reprogramming M2-like macrophages to the tumoricidal M1-like phenotype; (3) inhibiting the crosstalk between TAMs and tumor cells (Figure 3).

### 5.1. Depleting Total TAM Count

Lower macrophage count is considered a better prognostic indicator in PCa biopsy specimens. A study reported that the incidence of lymph node and bone metastasis in mice containing PC-3MM2 cells declined after macrophage abrogation induced by clodronate liposome. Consistent with these findings, macrophage depletion extended survival during ADT [127]. However, this attempt may be unsuccessful in clinical trials, largely because of severe, off-target side effects. Since its distinct marks or peripheral atmosphere, selective growth suppression of M2-subsets aids in prostate tumor regression and reduces metastatic potential in pre-clinical models.

As mentioned above, AR activation is responsible for M2-like differentiation. Compared with PTEN^+/−^ mice, prostate size remarkably decreased in genetic background mice with macrophage AR knockout (MARKO) and PTEN^+/−^, suggesting that AR-deficient macrophages can attenuate PTEN deficiency–induced prostate carcinogenesis. A study demonstrates that a selective AR degradation enhancer, ASC-J9® negates the pro-survival activity of TAMs and re-models TME towards an antitumoral immunity, providing a potential drug target for restraining early PIN development. Once a neoplasm has started, several approaches to androgen deprivation, like surgical castration, reversely modulate the accumulation of M2-polarized TAMs, which can be undermined by metformin through inducing COX2/PGE2 downregulation. Against this backdrop, a rational combination of metformin with ADT was proposed to augment the durable response in cancer patients.

Due to the tumor-homing ability of TAMs, chemokine-chemokine receptor blockers may display potential for therapeutics in the future. As described previously, RON overexpression in PCa cells enhances CCL2 production for macrophage recruitment and RON-overexpressing tumors alter macrophage state to drive growth under androgen-deprived conditions. It is therefore plausible that combining RON inhibition with macrophage depletion promotes CRPC sensitization to ADT [169]. Coincidentally, anti CCR2 antagonist could reduce the side effects induced by ADT, although single antibody targeting CCL2 failed in a phase II clinical trial [170]. Preclinical evidence suggests that blocking the CSF1/CSF1R axis effectively prevents the influx of TAMs and weakens their pro-tumorigenic influence. Despite having negligible effects on prostate tumor growth in vitro, the selective CSF1-R inhibitor, PLX3397, can alone cause a significant reduction of myeloid-derived suppressor cells (MDSCs) and F4/80^+^ macrophages in primary tumor sites. With this concern in mind, PLX3397 may helpfully improve prognosis when combined with other regimens like docetaxel and irradiation [171]. At the molecular level, CSF1-R blockade impedes radiotherapy-induced Arg1, CSF-1, and MMP expression, thus preventing the acquired resistance mediated by M2-polarized macrophages [30].

Knock-down of TR4 nuclear receptors suppresses the macrophage infiltration and consequent malignant invasion and metastasis by altering the TIMP-1/MMP2/MMP9 signals, which indicates that developing small molecule inhibitors targeting TR4 represents a feasible strategy against PCa. Notably, the difference between M1 and M2 macrophages may be utilized to achieve differentiated strike. Due to higher expression of CD115 in M2 phenotype than M1 population, trabectedin preferentially reduces the amount of M2-like macrophages by targeting CD115^+^ cells to rehabilitate the bone microenvironment, eventually leading to lessened tumor burden in the skeleton [172]. Intra-tumoral invariant natural killer T (iNKT) cells also remodel the antitumor microenvironment. After iNKT cells are transferred into PCa-bearing mice, selective killing of M2 macrophages and M1-subtype survival would happen via cooperative CD1d, CD40, and Fas engagement [173]. Acidic TME is expected to be exploited for tumor-specific imaging and therapy. The increased intra-tumoral pH had no significant difference in myeloid cell infiltration, but it cut off M2-like TAM activation caused by prostate tumors. More prominently, pH-responsive peptides or pH-sensitive nano-systems can target-specific acidic milieu rich in macrophages to improve the total efficacy. Delivery of STAT6 inhibitor decorated into a nanocarrier with a pH-sensitive PEG outer layer that only sheds in the acidic TME diminishes the number of TAMs in tumor tissues, whereas avoiding regulation of M2-subpopulation in healthy organs with neutral pH [174].

### 5.2. Reprogramming M2-Like Macrophages to the Tumoricidal M1-Like Phenotype

Recently, the potential of reprogrammed macrophage subsets has been explored. Injection of M1-derived exosome-mimetic nanovesicles that skews M2 towards an immunocompetent profile resulted in smaller tumor size in vivo, and potentiated antitumor efficacy of immune checkpoint inhibitor, like anti-PD-L1 antibody [175]. Macrophage polarization is not fixed, and domesticating TAMs to reverse their pro-tumoral properties provides a therapeutic window. Hyperbaric oxygen therapy is greatly anticipated. It either modifies the hypoxic microenvironment via an increased supply of oxygen or induces ROS over-production, which reduces the number and activity of M2 macrophages [176]. The administration of zoledronic acid (ZA) boosts the production of Th-1 cytokines (IL-12 and poly: C) that re-educated TAMs towards M1-type to suppress primary tumor growth and spontaneous lung metastasis, which has been applied for treating symptomatic skeletal lesion [177]. TLR agonists, like CpG ODNs, enhance cellular phagocytosis by repolarizing M2-M1 macrophages [66]. Likewise, paclitaxel alters the signature of TAMs into an M1-like profile in a TLR4-dependent manner, thus disrupting tumor promotion [178]. Exosomes have recently emerged as attractive natural nano-sized vesicles for drug delivery, due to their excellent biocompatibility and potential capacity to express targeting ligands [179]. The IFN-*γ* fusion protein was anchored in the PCa cell–derived exosome to prepare the IFN-*γ*-exosomal vaccine. Notably, it increases the quantity of M1 macrophages and enhances their ability to engulf RM-1 cell-derived exosomes, thereby clearing the regulatory effects of the latter. Pharmacological inhibition of cholesterol metabolism is also beneficial to M2 reprogramming. In the cellular study, Simvastatin adjusts the M2-M1 phenotypic transition of murine BMDMs via inhibition of LXR/ABCA1 responsible for cholesterol homeostasis, with TNF-*α* increase and TGF-*β* decrease, ultimately overcoming EMT-induced chemoresistance [180].

However, several successful immunotherapies for PCa are highly dependent on the preexistence of macrophages in tumor sites. The principal effect of an adenoviral vector-encoding murine IFN-*β* on PC-3MM2 growth inhibition is indirectly influencing other host cells of the microenvironment. At present, repolarization of M2-like TAMs is regarded as a requirement for carcinostatic activities of IFN-*β*. It could induce a detectable increase in iNOS-positive cells and reduce levels of M2-associated molecules responsible for angiogenesis and tumor invasion [181]. Virulizin also had a favorable toxicity profile in various human tumor xenograft models including PCa, whereas whole-body loss of macrophages compromised its anti-cancer effects. Further analysis suggested that Virulizin formed a niche in the TME that attenuated tumor progression by reversing TAMs into a pro-inflammatory subtype with higher TNF-*α* levels. More significantly, Virulizin increases IL-12*β* production in M1-like macrophages, thus enhancing NK cells–mediated cytotoxicity against PCa [182].

Furthermore, researchers observed that when PCa cells were present, M1 phenotype could not be fully restored even with M1-like cytokine stimulation, usually accompanied by diminished cytotoxicity. This finding implies that attempts to repolarize prostate TAMs will be sufficiently effective with concomitantly destroying adjacent tumor cells [183].

### 5.3. Inhibiting the Crosstalk between TAMs and Tumor Cells

Malignant cells readily cooperate with TAMs to aggravate tumor evolvement by forming a vicious cycle. The protective effects of dihydroisotanshinone I (DT) against PCa are just achieved by targeting their crosstalk via inhibition of the CCL2/STAT3 axis [184]. The majority of TAMs infiltrating PTEN-null PCa usually expressed the CXCR2 receptor; therefore, pharmacological blockade of the CXCR2 re-educated TAMs toward a TNF*α*-releasing pro-inflammatory phenotype to induce senescence and tumor inhibition. Meanwhile, it should be noted that the employment of CXCR2 antagonist needs to take into account the level of PTEN in the tumors, as these tumor cells with Pten deletion upregulate TNFR1 [185]. TAMs secrete IL-6, whereas the inhibition of the IL-6/STAT3 axis re-sensitizes PCa cells to paclitaxel. NF-*κ*B signaling also plays a crucial role in the action of this paracrine loop. Within the TME, cytokines derived from TAMs increase the activity of NF-*κ*B in the neoplastic population, which in turn stimulates macrophage infiltration via increased CSF-1 to promote prostate tumorigenesis. However, somatostatin derivate (smsDX) dramatically counteracts these effects by inhibiting the NF-*κ*B pathway. In addition, smsDX could dampen PCa-metastatic potential provoked by M2-like macrophages by binding with its somatostatin receptor 1/2 (SSRT1/2) [186]. Enjoyably enough, the polarization of THP-1 cells co-cultured with PC-3 cells is skewed to M1-like macrophages upon etoposide treatment, followed by an increase in etoposide-induced apoptosis of tumor cells [187]. Resolvin D2 (RVD2) alone has no effects on the proliferation of PCa cells, whereas attenuating their growth rate in a Transwell model. Further analysis shows that it diminished excretive growth factors (VEGFa and EGF) in THP‐1 cells, suggesting that RVD2 exerts cytotoxic functions through intervening by cell-to-cell wireless communication [67]. S-nitrosoglutathione (GSNO), an NO donor, impedes CRPC growth in the murine model. Studies report that the inhibitive action of GSNO on CRPC is targeting the TME but is not cell-autonomous. Compared to PBS-treated mice, GSNO suppressed the generation of various cytokines, especially M-CSF and BMP-6. As a result, the M1/M2 ratio was increased. On the other hand, p-ERK-mediated VEGF in macrophages was inhibited following therapy with GSNO, indicating a disruptive effect of NO on TAM activity [188].

The subcellular mechanism underlying the cholesterol exchange between tumor cells and macrophages has yet to be identified. However, the potent agonist of liver X receptor *β* (LXR*β*), RGX-104 limits their communication by preventing cholesterol metabolism. Therefore its application is expected to extend survival after ADT. A recent report shows statins, known to decrease systemic cholesterol levels, significantly improve the benefits of ADT in patients [189]. These agents reduced the availability of circulating cholesterol for ingestion by TAMs and its transfer towards neighboring tumor cells during ADT, possibly delaying the onset of CRPC. Similarly, treatment with PBP10, an inhibitor of lipid molecule lipoxin A4 (LXA4), abolished the role of PCa cell-derived LXA4 in M2 phenotype transformation by inhibiting METTL3/STAT6 [190].

## 6. Conclusions and Future Perspectives

In response to certain surrounding stimuli, macrophages are recruited into the TME and convert into TAMs. It is increasingly clear that such TME preferentially drives macrophages to undergo M2-like polarization, and the altered macrophages play a vital role in influencing the process of prostate tumor growth, metastasis, and therapeutic resistance. Since TAMs occupy the large number of intratumorally infiltrating immune cells, more attention would be paid to develop novel immunotherapies directly targeting TAMs or their functional mediators for improved treatment efficacy.

To expedite clinical translation, we need to clarify several questions in future directions. Which histological types of patients are suitable for TAM-directed therapy? More evidence indicated that relative contributions of TAM-subtypes in these populations need to be considered for precision medicine. Could the patients achieve benefits from long-term M2-M1 repolarization treatment? More importantly, which transcription factors are involved in phenotypic reversibility? How do epigenetic factors modulate gene profiles of TAMs, and are they firmly inherited? Unfortunately, the detailed connection between TAMs and tumor cells has not been fully determined, which remains informative to pursue. Furthermore, enhancing our knowledge on the origin and functions of TAM subsets in the tumor milieu will contribute to tapping TAMs-targeted therapeutic potential to the full as adjuvant antitumor strategies.

## Data Availability

The data used to support the findings of this study can be obtained from Chenglin Han and Xiaming Liu upon request.
